# Copper/Zinc Ratio as a Predictor of Chronic Kidney Disease Incidence in Type 2 Diabetes: The Asahi Diabetes Complications Study

**DOI:** 10.7759/cureus.88573

**Published:** 2025-07-23

**Authors:** Toshiko Takao, Hiroyuki Yanagisawa, Machi Suka, Yoko Yoshida, Mitsuhiko Noda, Masato Kasuga

**Affiliations:** 1 JR East Health Promotion Center, East Japan Railway Company, Tokyo, JPN; 2 Division of Diabetes and Metabolism, The Institute of Medical Science, Asahi Life Foundation, Tokyo, JPN; 3 Department of Public Health and Environmental Medicine, The Jikei University School of Medicine, Tokyo, JPN; 4 Ichikawa Hospital, International University of Health and Welfare, Ichikawa, JPN; 5 Department of Endocrinology and Diabetes, Saitama Medical University, Moroyama, JPN

**Keywords:** chronic kidney disease, copper/zinc ratio, inflammation, soluble tumor necrosis factor-α receptor 1 (stnfαr1), type 2 diabetes

## Abstract

Aim

This study aims to evaluate the associations between the copper/zinc (Cu/Zn) ratio and inflammatory biomarkers and the incidence of chronic kidney disease (CKD) in individuals with type 2 diabetes and to validate our previous cross-sectional study using the baseline data of this study.

Methods

We conducted a prospective, observational study of 416 individuals with type 2 diabetes without CKD. We used multivariable Cox proportional hazard models to determine the HRs for CKD incidence. The Cu/Zn ratio and soluble tumor necrosis factor-α receptor 1 (sTNFαR1) concentrations (pg/mL) were analyzed as continuous variables and as categories classified according to each cutoff value for detecting CKD. The high-sensitivity C-reactive protein (hsCRP) concentrations between these categories were compared.

Results

CKD was identified in 165 participants. The Cu/Zn ratio and sTNFαR1 concentrations were identified as significant predictors, independent of each other, after full adjustment (P = 0.048 and P = 0.006, respectively). Compared to the Cu/Zn <1.281 and sTNFαR1 <1081 group, the HRs for the CKD incidence were significantly higher in the Cu/Zn <1.281 and sTNFαR1 ≥1081 group (HR 2.06, 95% CI 1.34-3.26) and even higher in the Cu/Zn ≥1.281 and sTNFαR1 ≥1081 group (3.29, 1.97-5.50) after full adjustment. The hsCRP concentrations were significantly highest in the Cu/Zn ≥1.281 and sTNFαR1 ≥1081 group compared with the other three groups (all P < 0.05) and were significantly higher in the Cu/Zn ≥1.281 and sTNFαR1 <1081 group and in the Cu/Zn <1.281 and sTNFαR1 ≥1081 group compared with those in the Cu/Zn <1.281 and sTNFαR1 <1081 group (both P < 0.05).

Conclusions

The Cu/Zn ratio and sTNFαR1 concentrations are independent predictors of the incidence of CKD in individuals with type 2 diabetes. Furthermore, under elevated sTNFαR1 concentrations, an increase in the Cu/Zn ratio may further aggravate inflammation and accelerate the incidence of CKD in individuals with type 2 diabetes. These new findings are supported by the results from our previous cross-sectional study.

## Introduction

Three interrelated pathophysiological processes - metabolic, hemodynamic, and inflammatory/fibrotic - cause the development and progression of chronic kidney disease (CKD) in individuals with type 2 diabetes [1-4]. Recently, the understanding of the pathogenesis of CKD in individuals with diabetes has advanced, leading to the recognition that inflammation is a pivotal underlying mechanism of kidney dysfunction [3]. Individuals with type 2 diabetes still have a residual risk for CKD, despite current therapies. Increasing evidence has shown that inflammation/fibrosis is a residual risk in the pathogenesis and progression of CKD [5-7]. Oxidative stress is closely associated with inflammation/fibrosis through the activation of signaling pathways and transcription factors that stimulate the production of pro-inflammatory cytokines and fibrogenic factors [1].

A substantial body of research has indicated that zinc (Zn) is essential for the regulation of cytokine expression, the suppression of inflammation, and the activation of antioxidant enzymes that scavenge reactive oxygen species, thereby contributing to the reduction of oxidative stress [8]. Zn deficiency has been shown to exacerbate renal inflammation, oxidative stress, and fibrosis induced by diabetes [9]. Zn is essential for the synthesis, storage, and release of insulin. This suggests that Zn plays a pivotal role in the progression of type 2 diabetes and its complications [8]. In addition, excess copper (Cu) under inflammatory conditions triggers oxidative stress [10]. Elevated serum Cu concentrations can lead to the deposition of Cu in the kidneys, resulting in nephrotoxicity involving interstitial damage, which can progress to renal dysfunction [11]. Cu imbalance can impair antioxidant homeostasis and contribute to the progression of diabetes-related complications [11]. A Mendelian randomization study reported that elevated circulating Cu concentrations determined genetically might be a causative risk for CKD and could potentially lower the estimated glomerular filtration rate (eGFR), leading to a rapid decline in renal function [12].

Compensatory mechanisms strictly regulate serum concentrations of Zn and Cu, stabilizing them within certain nutritional intake ranges. However, mechanisms exist that decrease serum Zn concentrations and increase serum Cu concentrations in the presence of inflammatory conditions. Therefore, an increase in the Cu to Zn (Cu/Zn) ratio is a feature common to several age-related chronic diseases [13]. We previously conducted a cross-sectional study using baseline data from this prospective cohort study. The results showed that under a starting signal for inflammation of elevated concentrations of serum soluble tumor necrosis factor-α receptor 1 (sTNFαR1), an increase in the Cu/Zn ratio may aggravate inflammation and is linked to a high prevalence of diabetic kidney disease in individuals with type 2 diabetes [14].

This prospective cohort study aimed to evaluate the associations of the Cu/Zn ratio and sTNFαR1 concentrations with the incidence of CKD in individuals with type 2 diabetes. We also aimed to validate the results of the baseline cross-sectional study.

## Materials and methods

Study participants

**Figure 1:**
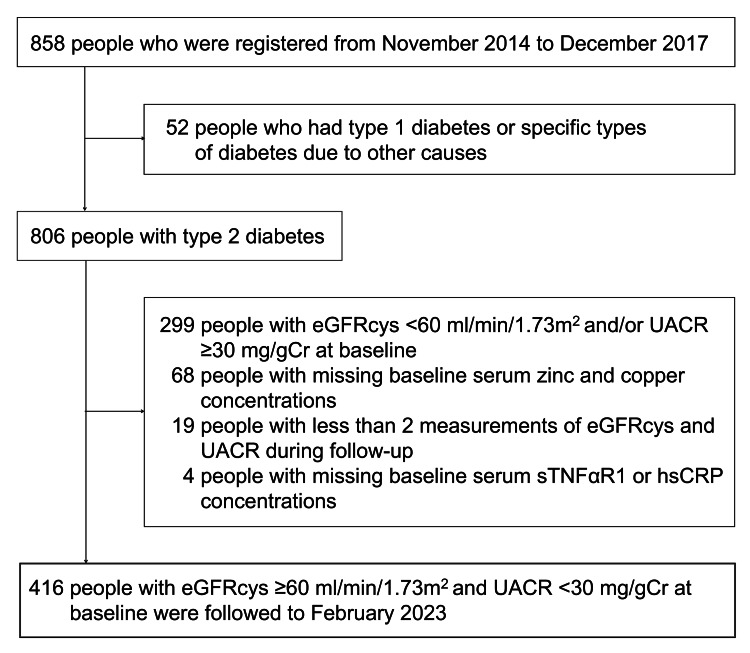
Flow diagram of the study participants included in the analyses eGFR: estimated glomerular filtration rate using cystatin C; hsCRP: high-sensitivity C-reactive protein; sTNFαR1: soluble tumor necrosis factor-α receptor 1; UACR: urinary albumin-to-creatinine ratio

The present study used data from the Asahi Diabetes Complications Study (Asahi Study). The Asahi Study is a prospective observational cohort study focusing on the development and progression of diabetic complications in individuals with diabetes who attended our outpatient clinic specializing in diabetes care. Study participants were recruited from November 2014 to December 2017. The exclusion criteria for participants have been described previously [14]. Figure 1 shows a flow diagram of the participants enrolled in the analyses. A total of 858 individuals were registered in this study, 806 of whom had type 2 diabetes. Of these 806 individuals, 435 exhibited an eGFR using cystatin C (eGFRcys) of ≥60 mL/min/1.73 m² and a urinary albumin-to-creatinine ratio (UACR) of <30 mg/g creatinine at baseline. They also had baseline measurements of serum Zn and Cu concentrations and inflammatory markers, including sTNFαR1 and high-sensitivity C-reactive protein (hsCRP). Of these 435 individuals, 19 were excluded due to the presence of a solitary eGFRcys and UACR measurement during the follow-up period. Consequently, at baseline, 416 individuals (336 men and 80 women) were followed to February 2023. The follow-up methods were clinic visits and record linkage, not telephone or postal communication.

Measurement of the serum Cu/Zn ratio and inflammatory biomarkers

At baseline, blood samples for the measurement of Zn and Cu concentrations were obtained from Asahi Study participants who attended the outpatient clinic by 10:00 AM following a night of fasting. A colorimetric method was used to determine serum Zn and Cu concentrations [15,16]. Subsequently, the serum Cu/Zn ratio was calculated.

Concentrations of sTNFαR1 have been observed to demonstrate enhanced stability in comparison to tumor necrosis factor-α concentrations, exhibiting prolonged periods of elevated levels. Furthermore, elevated concentrations of sTNFαR1 have been demonstrated to serve as a reliable indicator of the activation of the tumor necrosis factor-α system [17,18]. Concentrations of sTNFαR1 were determined by an enzyme-linked immunosorbent assay (Human TNF RI/TNFRSF1A Quantikine ELISA Kit; R&D SYSTEMS, Minneapolis, MN, USA). The detection range was 78.125 to 5000 pg/mL. Concentrations of hsCRP were determined using immunoturbidimetry. The measurement of these inflammatory biomarkers was conducted in the morning following a night of fasting.

Assessment of CKD

The UACR was measured annually using a turbidimetric immunoassay. Concentrations of cystatin C, which is a biomarker of renal function, were measured annually using the colloidal gold agglutination method [19]. Cystatin C has emerged as an alternative filtration biomarker, exhibiting a greater propensity to demonstrate linear risk associations in comparison with creatinine [20-22]. Consequently, cystatin C is regarded as a superior biomarker, with the capacity to reliably facilitate risk classification in clinical settings [22]. Empirical evidence has demonstrated the efficacy of the eGFRcys in enhancing the association between eGFR categories and the risks of mortality and end-stage renal disease across diverse demographics, in comparison with the utilization of creatinine-based eGFR [23]. The eGFRcys was computed according to the formula endorsed by the Japanese Society of Nephrology [24]. CKD was defined as a UACR ≥30 mg/g creatinine and/or an eGFRcys <60 mL/min/1.73 m². The combination of the eGFRcys and the UACR provides a significant improvement in risk classification [22].

Collection of clinical and biochemical data

An evaluation of the subjects’ height and weight was conducted, and the BMI of each was subsequently computed. Their BP was measured once in a sitting posture by a trained medical technologist with an electronic sphygmomanometer (BP-10; OMRON, Kyoto, Japan). The duration of diabetes was confirmed from the attending physician's medical records. Information regarding smoking history and alcohol consumption was collected by interview sheets completed by the study participants. Individuals who consumed alcohol at levels ≥40 g/day among men and ≥20 g/day among women were categorized as drinkers [25]. Following a night of fasting, blood samples were collected in the morning. Glycated hemoglobin (HbA1c) concentrations were assayed with an analyzer (HLC-723G8, HLC-723G11; TOSOH, Tokyo, Japan) using a high-performance liquid chromatography method. The concentrations of serum high-density lipoprotein cholesterol (HDLC) and serum low-density lipoprotein cholesterol (LDLC) were assayed by a direct method, whereas the concentrations of serum triglyceride (TG) were assayed by an enzymatic method.

Ethics

The Ethics Committee of the Institute of Medical Science, Asahi Life Foundation, approved the research protocol (approval 07501). The protocol was reviewed and confirmed to be following the Japanese government's Ethical Guidelines for Medical and Health Research Involving Human Subjects, which are in alignment with the provisions of the Declaration of Helsinki. All of the enrolled participants provided written informed consent. The study/trial registration and approval date is November 26, 2014, with registration number UMIN000015744.

Statistical analysis

A comparative analysis of baseline clinical characteristics was conducted between participants who did and those who did not develop CKD by use of Student’s t-test, the Wilcoxon rank-sum test, the χ² test, and Fisher’s exact test. The UACR and hsCRP, sTNFαR1, and TG concentrations showed a skewed distribution. Therefore, these variables were converted to natural logarithms for analysis. Cases with missing data were simply deleted (handled by complete case analysis or listwise deletion).

The discriminating power of the Cu/Zn ratio and sTNFαR1 concentration for the detection of CKD was assessed by using the area under the receiver operating characteristic (ROC) curve (AUC) by univariable logistic regression models. The observation period was set to approximately five years. The cutoff values of these biomarkers for the detection of CKD incidence were derived from Youden's index using ROC analyses.

Multivariable Cox proportional hazard models were applied to calculate HRs for the incidence of CKD related to the Cu/Zn ratio and sTNFαR1 concentrations, which were treated as continuous variables and as the categories dichotomized by each cutoff point. The Cu/Zn ratio was included as a continuous variable in Model 1. Concentrations of sTNFαR1 were included as a continuous variable in Model 2. In Model 3, both of these variables were included simultaneously for mutual adjustment. The dichotomized Cu/Zn ratio category at its cutoff value was included in Model 4. The dichotomized sTNFαR1 category at its cutoff value was included in Model 5. In Model 6, both of these dichotomized categories were included simultaneously for mutual adjustment. In Model 7, four categories classified by each cutoff value for the Cu/Zn ratio and sTNFαR1 concentrations were included. Three sets of covariates were included in these models. Age and gender were the first set. The second set comprised age, sex, baseline eGFRcys, baseline UACR (ln-transformed), systolic BP, BMI, diabetes duration, HbA1c concentrations, LDLC concentrations, HDLC concentrations, TG concentrations (ln-transformed), and smoking status. The third set included the second set of variables plus insulin, sodium glucose co-transporter 2 inhibitor (SGLT2i), glucagon-like peptide-1 receptor agonist (GLP-1RA), renin-angiotensin-aldosterone system (RAAS) inhibitor, and statin use.

Concentrations of hsCRP (ln-transformed), which are an indicator of systemic inflammation, were compared between these four categories classified according to each cutoff value for the Cu/Zn ratio and sTNFαR1 concentrations using an analysis of variance (Tukey-Kramer’s test).

All statistical analysis was performed with SAS software version 9.4 (SAS Institute, Cary, NC, USA). Statistical significance was defined as a two-tailed P-value <0.05.

## Results

CKD was identified in 165 participants (39.7%). Of these 165 participants, 52 (31.5%) progressed to CKD stage 3a while maintaining normoalbuminuria. The remaining 113 participants (68.5%) developed microalbuminuria; of these, only two (1.2%) had both microalbuminuria and progression to CKD stage 3a. The median (IQR) follow-up period until the onset of CKD or end of follow-up was 4.9 (3.0-5.1) years. The loss to follow-up rate was 10.1%.

Comparison of baseline clinical characteristics and biomarkers between participants who did and did not develop CKD

The baseline clinical features of all participants and those who did and did not progress to CKD are listed in Table 1.

**Table 1:** Baseline clinical characteristics of all participants and participants who did or did not progress to CKD Values are n (%), mean ± SD, or median (IQR). ^*^ Alcohol intake ≥40 g/day for men and ≥20 g/day for women. ^†^ Calculated using Student’s t-test. ^‡^ Calculated using χ² test. ^§^ Calculated using Fisher’s exact test. ^||^ Calculated using the Wilcoxon rank sum test. CKD: chronic kidney disease; Cu: copper; eGFRcreat: estimated glomerular filtration rate using creatinine; eGFRcys: estimated glomerular filtration rate using cystatin C; GLP-1RA: glucagon-like peptide-1 receptor agonist; Hb: hemoglobin; HbA1c: glycated hemoglobin; HDLC: high-density lipoprotein cholesterol; hsCRP: high-sensitivity C-reactive protein; LDLC: low-density lipoprotein cholesterol; OAD: oral antidiabetes drugs; RAAS: renin-angiotensin-aldosterone system; sTNFαR1: soluble tumor necrosis factor-α receptor 1; SGLT2i: sodium glucose co-transporter 2 inhibitors; TG: triglycerides; UA: uric acid; UACR: urine albumin-to-creatinine ratio; Zn: zinc

Variable	Total (n = 416)	Participants who progressed to CKD (n = 165)	Participants who did not progress to CKD (n = 251)	Statistic value	P-value
Male sex	336 (80.8)	134 (81.2)	202 (80.5)	χ² = 0.0345	0.85^‡^
Age (years)	63.4 ± 9.0	65.3 ± 9.3	62.2 ± 8.7	t = 3.41	0.001^†^
Diabetes duration (years)	15.7 (8.9-22.3)	16.4 (9.2-22.9)	14.8 (8.4-21.8)	Z = 1.5775	0.11^||^
BMI (kg/m²)	24.3 ± 3.6	24.9 ± 3.9	23.8 ± 3.3	t = 3.07	0.002^†^
Systolic BP (mmHg)	129.2 ± 12.5	131.2 ± 12.2	127.9 ± 12.5	t = 2.69	0.008^†^
Diastolic BP (mmHg)	76.3 ± 8.6	76.3 ± 9.0	76.2 ± 8.4	t = 0.05	0.96^†^
HbA1c (%) (mmol/mol)	6.9 ± 0.8 (52 ± 8)	7.0 ± 0.8 (53 ± 9)	6.9 ± 0.7 (52 ± 8)	t = 0.50	0.62^†^
LDLC (mmol/L)	2.70 ± 0.59	2.64 ± 0.54	2.74 ± 0.62	t = 1.62	0.11^†^
HDLC (mmol/L)	1.51 ± 0.40	1.48 ± 0.42	1.53 ± 0.38	t = 1.41	0.16^†^
TG (mmol/L)	1.05 (0.75-1.56)	1.14 (0.80-1.74)	1.00 (0.72-1.51)	Z = 1.9140	0.056^||^
Hb (g/L)	144.8 ± 11.9	143.6 ± 12.4	145.5 ± 11.5	t = 1.59	0.11^†^
UA (umol/L)	325.5 ± 69.6	332.9 ± 70.9	320.7 ± 68.5	t = 1.76	0.079^†^
Creatinine (μmol/L)	68.4 ± 13.1	70.0 ± 14.2	67.4 ± 12.2	t = 1.88	0.061^†^
eGFRcreat (mL/min/1.73m²)	76.9 ± 14.3	75.0 ± 15.4	78.1 ± 13.4	t = 2.15	0.032^†^
Cystatin C (mg/L)	0.88 ± 0.13	0.92 ± 0.13	0.85 ± 0.12	t = 5.54	<0.0001^†^
eGFRcys (mL/min/1.73 m²)	85.0 ± 15.4	80.0 ± 15.3	88.2 ± 14.6	t = 5.56	<0.0001^†^
UACR (mg/g creatinine)	7.9 (4.9-12.9)	12.3 (7.2-19.3)	6.4 (4.1-9.4)	Z = 8.7616	<0.0001^||^
sTNFαR1 (pg/mL)	1080 (954-1240)	1170 (1030-1310)	1020 (931-1180)	Z = 5.6813	<0.0001^||^
hsCRP (ng/mL)	415 (207.5-898)	442 (215-1000)	396 (200-847)	Z = 1.2933	0.20^||^
Cu (μg/dL)	97.2 ± 15.6	97.6 ± 14.3	97.0 ± 16.5	t = 0.42	0.67^†^
Zn (μg/dL)	85.5 ± 11.3	83.3 ± 10.8	86.9 ± 11.4	t = 3.24	0.001^†^
Cu/Zn	1.157 ± 0.242	1.193 ± 0.240	1.133 ± 0.240	t = 2.47	0.014^†^
Current smoker	85 (20.4)	36 (21.8)	49 (19.5)	χ² = 0.3229	0.57^‡^
Alcohol intake^*^	51 (12.3)	20 (12.1)	31 (12.4)	χ² = 0.0049	0.94^‡^
Use of antidiabetic agents					
OAD	291 (70.0)	121 (73.3)	170 (67.7)	χ² = 1.4876	0.22^‡^
SGLT2i	37 (8.9)	12 (7.3)	25 (10.0)	χ² = 0.8873	0.35^‡^
GLP-1RA	33 (7.9)	11 (6.7)	22 (8.8)	χ² = 0.6002	0.44^‡^
Insulin	90 (21.6)	35 (21.2)	55 (21.9)	χ² = 0.0288	0.87^‡^
Use of antihypertensive agents					
RAAS inhibitors	180 (43.3)	98 (59.4)	82 (32.7)	χ² = 28.9661	<0.0001^‡^
Calcium-channel blockers	128 (30.8)	70 (42.4)	58 (23.1)	χ² = 17.4387	<0.0001^‡^
α-, β-, αβ-blockers	25 (6.0)	16 (9.7)	9 (3.6)	χ² = 6.5827	0.010^‡^
Diuretics	12 (2.9)	10 (6.1)	2 (0.8)		0.002^§^
Use of lipid-lowering agents					
Statins	219 (52.6)	85 (51.5)	134 (53.4)	χ² = 0.1398	0.71^‡^
Fibrates	17 (4.1)	7 (4.2)	10 (4.0)	χ² = 0.0170	0.90^‡^

The CKD group was significantly older (P = 0.001), and they had higher systolic BP (P = 0.008), BMI (P = 0.002), UACR (P < 0.0001), and Cu/Zn ratio (P = 0.014). They also had significantly higher concentrations of cystatin C (P < 0.0001) and sTNFαR1 (P < 0.0001) compared with the non-CKD group. The CKD group had significantly lower eGFR using creatine (P = 0.032), eGFRcys (P < 0.0001), and Zn concentrations (P = 0.001) compared with the non-CKD group. The CKD group had higher percentages of people using antihypertensive agents, namely RAAS inhibitors (P < 0.0001), calcium channel blockers (P < 0.0001), α-, β-, and αβ-blockers (P = 0.010), and diuretics (P = 0.002), compared with the non-CKD group. The frequency of use of SGLT2i and GLP-1RA was still low at baseline. There was no significant difference between the CKD and non-CKD groups in the proportion of users of SGLT2i or GLP-1RA.

Optimal cutoff values of the Cu/Zn ratio and sTNFαR1 concentrations for detecting the incidence of CKD

**Figure 2:**
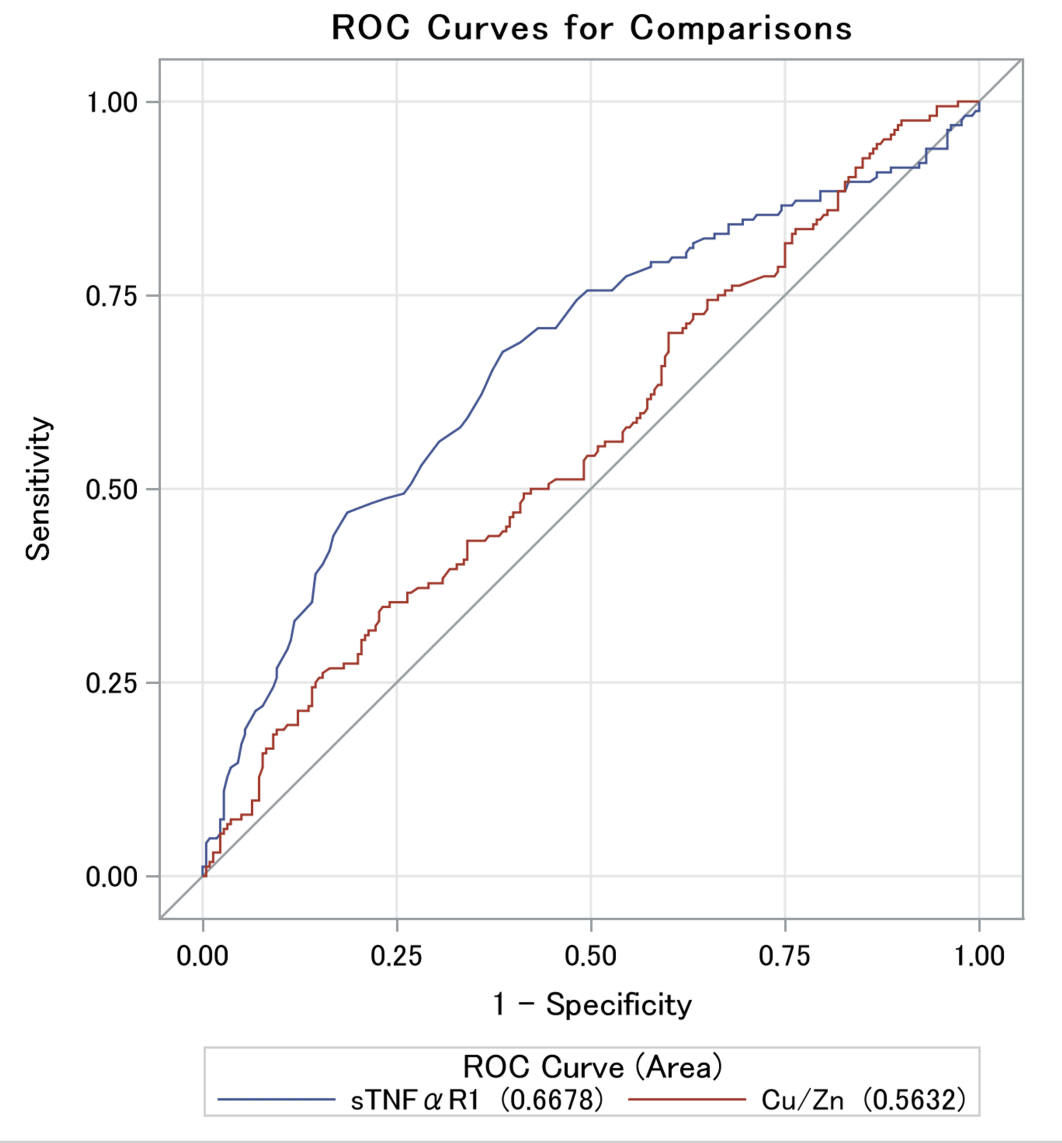
ROC curves of sTNFαR1 concentrations and the Cu/Zn ratio for detecting the incidence of CKD CKD: chronic kidney disease; Cu: copper; ROC: receiver operating characteristic; sTNFαR1: soluble tumor necrosis factor-α receptor 1; Zn: zinc

The discriminatory abilities of the Cu/Zn ratio and sTNFαR1 concentrations for detecting the incidence of CKD were determined using ROC curves (Figure 2).

The AUC (95% CI) and optimal cutoff values were 0.563 (0.505-0.621) and 1.281 for the Cu/Zn ratio and 0.668 (0.612-0.724) and 1081 pg/mL for sTNFαR1 concentrations, respectively. The Cu/Zn ratio had a sensitivity of 34.8%, specificity of 76.8%, positive predictive value of 52.8%, and negative predictive value of 61.2%. Those for sTNFαR1 concentration were 67.7%, 61.4%, 56.6%, and 71.8%, respectively. The AUC for the sTNFαR1 concentrations was significantly greater compared with that for the Cu/Zn ratio (P = 0.011).

CKD incidence related to the Cu/Zn ratio and sTNFαR1 concentrations

Multivariable Cox regression analyses were implemented to ascertain the HRs for the incidence of CKD related to the Cu/Zn ratio and sTNFαR1 concentrations. These variables were examined as both continuous variables and categories classified by the respective cutoffs of the Cu/Zn ratio (1.281) and the sTNFαR1 concentration (1081 pg/mL), as detailed in Table 2.

**Table 2:** Multivariable Cox proportional hazard models for the incidence of CKD related to serum Cu/Zn ratio and sTNFαR1 concentrations The Cox proportional hazards models were used for analysis. ^*^ Adjusted for age, sex, eGFRcys at baseline, UACR (ln-transformed), BMI, systolic BP, diabetes duration, HbA1c levels, LDLC levels, HDLC levels, triglyceride levels (ln-transformed), and current smoking. ^†^ Further adjusted for use of insulin, use of an SGLT2i, use of a GLP-1RA, use of a renin-angiotensin-aldosterone system inhibitor, and use of a statin in addition to the above covariates. ^‡^ Classified by the cutoff value of a Cu/Zn ratio of 1.281. ^§^ Classified by the cutoff value of a sTNFαR1 level of 1081 pg/mL. Cu: copper; CKD: chronic kidney disease; eGFRcys: estimated glomerular filtration rate using cystatin C; GLP-1RA: glucagon-like peptide-1 receptor agonist; HbA1c: glycated hemoglobin; HDLC: high-density lipoprotein cholesterol; LDLC: low-density lipoprotein cholesterol; SGLT2i: sodium glucose co-transporter 2 inhibitor; sTNFαR1: soluble tumor necrosis factor-α receptor 1; UACR: urinary albumin-to-creatinine ratio; Zn: zinc

Model	Events/patients	Age and sex adjusted		Multivariable adjusted^*^		Full adjusted^†^	
		HR (95% CI)	P-value	HR (95% CI)	P-value	HR (95% CI)	P-value
Model 1							
Cu/Zn	165/416	1.97 (1.06-3.68)	0.033	2.40 (1.24-4.68)	0.01	2.33 (1.21-4.50)	0.012
Model 2							
sTNFαR1 (100 pg/mL increment)	165/416	1.26 (1.17-1.35)	<0.0001	1.16 (1.06-1.27)	0.0008	1.15 (1.06-1.26)	0.002
Model 3							
Cu/Zn	165/416	1.56 (0.84-2.91)	0.16	2.02 (1.04-3.94)	0.039	1.95 (1.01-3.80)	0.048
sTNFαR1 (100 pg/mL increment)	165/416	1.25 (1.16-1.34)	<0.0001	1.14 (1.05-1.25)	0.003	1.14 (1.04-1.24)	0.006
Model 4^‡^							
Cu/Zn ≥1.281	58/117	1.51 (1.08-2.11)	0.017	1.45 (1.03-2.04)	0.032	1.51 (1.07-2.13)	0.02
Model 5^§^							
sTNFαR1 ≥1081 pg/mL	111/206	2.52 (1.81-3.51)	<0.0001	2.10 (1.45-3.05)	<0.0001	2.17 (1.49-3.16)	<0.0001
Model 6							
Cu/Zn ≥1.281	58/117	1.57 (1.12-2.19)	0.009	1.50 (1.07-2.11)	0.019	1.51 (1.07-2.13)	0.02
sTNFαR1 ≥1081 pg/mL	111/206	2.56 (1.84-3.57)	<0.0001	2.16 (1.48-3.15)	<0.0001	2.18 (1.49-3.20)	<0.0001
Model 7							
Cu/Zn ≥1.281 and sTNFαR1 ≥1081 pg/mL	38/61	3.98 (2.49-6.37)	<0.0001	3.25 (1.95-5.42)	<0.0001	3.29 (1.97-5.50)	<0.0001
Cu/Zn <1.281 and sTNFαR1 ≥1081 pg/mL	73/145	2.37 (1.57-3.56)	<0.0001	1.96 (1.26-3.04)	0.003	2.09 (1.34-3.26)	0.001
Cu/Zn ≥1.281 and sTNFαR1 <1081 pg/mL	20/56	1.35 (0.77-2.39)	0.3	1.24 (0.69-2.20)	0.47	1.38 (0.77-2.48)	0.28
Cu/Zn <1.281 and sTNFαR1 <1081 pg/mL	34/154	1.00 (Reference)		1.00 (Reference)		1.00 (Reference)	

The covariates are provided in the Statistical analysis section. It was demonstrated in Models 1 and 2 that the Cu/Zn ratio and sTNFαR1 concentrations function as significant predictors of the incidence of CKD, following adjustment for age and sex (P = 0.033 and P < 0.0001, respectively), following multivariable adjustment (P = 0.010 and P = 0.0008, respectively), and following full adjustment (P = 0.012 and P = 0.002, respectively). In Model 3, the Cu/Zn ratio and sTNFαR1 concentrations were identified as significant predictors, independent of each other, following multivariable adjustment (P = 0.039 and P = 0.003, respectively) and following full adjustment (P = 0.048 and P = 0.006, respectively). In Models 4 and 5, the Cu/Zn ≥1.281 group and the sTNFαR1 ≥1081 pg/mL group were identified as significant predictors, following adjustment for age and sex (P = 0.017 and P < 0.0001, respectively), multivariable adjustment (P = 0.032 and P < 0.0001, respectively), and full adjustment (P = 0.020 and P < 0.0001, respectively). In Model 6, the Cu/Zn ≥1.281 group and the sTNFαR1 ≥1081 pg/mL group were identified as significant predictors independent of each other, following adjustment for age and sex (P = 0.009 and P < 0.0001, respectively), multivariable adjustment (P = 0.019 and P < 0.0001, respectively), and full adjustment (P = 0.020 and P < 0.0001, respectively). In Model 7, based on each cutoff of the Cu/Zn ratio and sTNFαR1 concentrations, participants were classified into four categories. The HRs for CKD relating to the four groups were then calculated (using the Cu/Zn <1.281 and sTNFαR1 <1081 pg/mL group as the reference). The HRs for CKD tended to be increased in the Cu/Zn ≥1.281 and sTNFαR1 <1081 pg/mL group after adjustment, but not significantly. The HRs for CKD were significantly increased in the Cu/Zn <1.281 and sTNFαR1 ≥1081 pg/mL group after adjusting for age and sex (P < 0.0001). This increase remained significant after multivariable adjustment (P = 0.003) and full adjustment (P = 0.001). The HRs were further significantly increased in the Cu/Zn ≥1.281 and sTNFαR1 ≥1081 pg/mL group after adjusting for age and sex (P < 0.0001). This increase remained significant after multivariable adjustment (P < 0.0001) and full adjustment (P < 0.0001).

**Figure 3:**
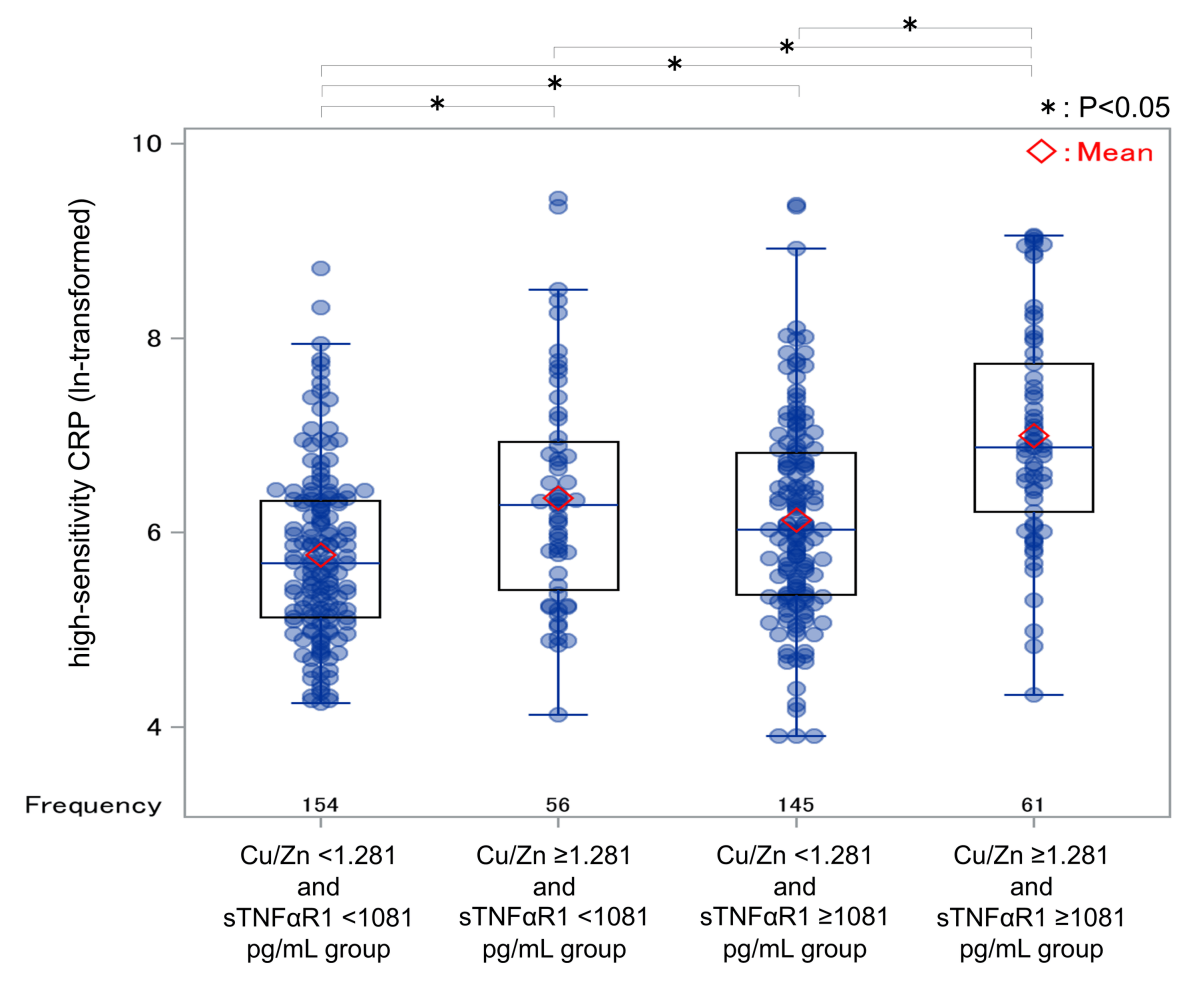
Comparison of hsCRP concentrations (ln-transformed) in the four groups classified by respective cutoff values of the Cu/Zn ratio and sTNFαR1 concentrations ANOVA (Tukey-Kramer’s test) was used for analysis. F-value = 22.02, P < 0.0001. Significant differences in hsCRP concentrations (all P < 0.05) were observed between all groups except for the Cu/Zn <1.281 and sTNFαR1 ≥1081 pg/mL group vs. the Cu/Zn ≥1.281 and sTNFαR1 <1081 pg/mL group. Cu: copper; hsCRP: high-sensitivity C-reactive protein; sTNFαR1: soluble tumor necrosis factor-α receptor 1; Zn: zinc

In Model 7, hsCRP concentrations (ln-transformed) were compared between these four groups (Figure 3).

A statistically significant difference in hsCRP concentrations was observed between these four groups (P < 0.0001). The Cu/Zn ≥1.281 and sTNFαR1 ≥1081 pg/mL group had the highest hsCRP concentrations, followed by the Cu/Zn <1.281 and sTNFαR1 ≥1081 pg/mL group, and the Cu/Zn ≥1.281 and sTNFαR1 <1081 pg/mL group. The Cu/Zn <1.281 and sTNFαR1 <1081 pg/mL group had the lowest hsCRP concentrations. Significant differences in hsCRP concentrations (all P < 0.05) were demonstrated between all groups except for the Cu/Zn <1.281 and sTNFαR1 ≥1081 pg/mL group vs. the Cu/Zn ≥1.281 and sTNFαR1 <1081 pg/mL group.

The Cu/Zn ratio exhibited a positive correlation with hsCRP concentrations (ln-transformed) (r = 0.376, P < 0.0001), while its correlation with sTNFαR1 concentrations (ln-transformed) was less significant (r = 0.105, P = 0.033). The correlation between hsCRP concentrations (ln-transformed) and sTNFαR1 concentrations (ln-transformed) was also not high (r = 0.298, P < 0.0001).

## Discussion

This study showed that the Cu/Zn ratio and sTNFαR1 concentrations were independent predictors of the incidence of CKD in individuals with type 2 diabetes. Furthermore, an increase in the Cu/Zn ratio exacerbated inflammation and accelerated the incidence of CKD when accompanied by an inflammatory initiating signal, characterized by elevated concentrations of serum sTNFαR1. These new findings were supported by those of our previously published cross-sectional study using baseline data [14]. In the present study, the Cu/Zn ratio and sTNFαR1 concentrations showed smaller AUCs and HRs and different cutoff values for the incidence of CKD compared with those observed in our previous cross-sectional study. However, the present results were derived from a prospective study, and we believe that they are reliable. Inflammation is known to be aggravated by the excessive production of reactive oxygen species inside the body. Superoxide dismutase (SOD) is thought to suppress inflammation by scavenging the reactive oxygen species, suggesting that reduced SOD activity may exacerbate inflammatory responses. The Cu/Zn ratio may affect SOD activity and regulate inflammation. In light of the potential for mitigating the residual risk of developing CKD, sustaining an adequate Cu/Zn ratio, possibly below 1.281, appears to be beneficial. Future research will hopefully lead to the determination of the optimal Cu/Zn ratio. The Cu/Zn ratio is a potential indicator of Zn deficiency severity [26]. This finding suggests the possibility of Zn supplementation as a means of reducing the residual risk of CKD progression in individuals with type 2 diabetes.

From CKD stages 1 to 3b, serum Zn concentrations showed a significant downward trend (P trend = 0.032), whereas Cu concentrations exhibited no significant trends in individuals with type 2 diabetes [27]. This previous report supports our findings. In the present study, serum Zn concentrations were significantly lower in individuals who developed CKD compared to those who did not. However, serum Cu concentrations did not differ significantly between individuals who developed CKD and those who did not. The Cu/Zn ratio appears to be a valuable marker in the evaluation of the onset and earlier stages of CKD in individuals with type 2 diabetes.

The Cu/Zn ratio has also been shown to be a predictor of mortality in the older population [28]. However, its role as a predictor or a biomarker of physical or functional performance appears to be the result of its strong correlation with inflammatory markers [28]. The present study showed that the Cu/Zn ratio predicted the incidence of CKD independent of the starting signal for inflammation (sTNFαR1), and it may act as an accelerating factor of the CKD incidence when accompanied by elevated serum sTNFαR1 concentrations. Although the Cu/Zn ratio is intimately linked to inflammation, this ratio appears to affect the chronic phase rather than the early stage. In a previous study, the Cu/Zn ratio was positively associated with systemic inflammation markers, including C-reactive protein concentrations and the erythrocyte sedimentation rate [29]. In the present study, the Cu/Zn ratio showed a weak positive correlation with hsCRP concentrations (ln-transformed), whereas its correlation with sTNFαR1 concentrations (ln-transformed) was little. An increase in the serum Cu/Zn ratio may enhance systemic inflammation by receiving elevated serum sTNFαR1 concentrations, an inflammatory initiating signal.

A previous study found that baseline serum sTNFαR1 concentrations were one of the strongest factors determining renal decline in individuals with type 1 diabetes [30]. This finding is consistent with our results in individuals with type 2 diabetes. This previous study also showed that 10% of people with normoalbuminuria and 35% of people with microalbuminuria had progressive renal decline [30]. In the present study, of the 165 individuals who developed CKD, 52 (31.5%) had reduced renal function while maintaining normoalbuminuria, and only two (1.2%) developed both microalbuminuria and reduced renal function. Therefore, in a certain proportion of type 2 diabetes individuals, early renal function decline begins during normoalbuminuria, before the development of microalbuminuria.

The present study’s strengths include its prospective observational cohort design, which enables the validation of the results of our baseline cross-sectional study [14]. In addition, sequential measurements of the eGFRcys facilitate a more accurate risk classification of CKD than that achieved using the creatinine-based eGFR in clinical settings. However, some limitations should be mentioned. First, our study participants were older and had a higher proportion of men. The evaluation of younger people and sex differences requires further examination. Second, the study participants consisted of people with type 2 diabetes who attended the clinic regularly and had relatively well-controlled glucose metabolism, BP, and lipids. Future research on subjects who do not meet these conditions is required. Third, the Cu/Zn ratio can be affected by diet, supplements, or medication use, but dietary data were not available. Fourth, missing data reduces statistical power and can lead to biased estimates. However, a listwise deletion method is known to generate unbiased estimates and conservative results when the assumption of missing completely at random is met. Fifth, genetic predisposition, an unmeasured confounder, was left unadjusted. Finally, our study was implemented at a single clinic in Japan, limiting its generalizability to other ethnicities.

## Conclusions

The Cu/Zn ratio and sTNFαR1 concentrations are independent predictors of the incidence of CKD in individuals with type 2 diabetes. Furthermore, under elevated serum sTNFαR1 concentrations as an inflammatory initiating signal, elevated Cu/Zn ratios may further aggravate inflammation and accelerate the incidence of CKD. These new findings are supported by the results from our previous cross-sectional study.

While serum sTNFαR1 concentration has been demonstrated to be an important risk factor for the incidence of CKD, to the best of my knowledge, this is the first clinical report on the Cu/Zn ratio in the incidence of CKD. Although the Cu/Zn ratio is not routinely measured in a clinical setting, this ratio deserves further attention. Measuring the Cu/Zn ratio in addition to serum sTNFαR1 concentrations as a risk marker allows for more accurate risk assessment. Individuals with both elevated serum sTNFαR1 concentrations and elevated Cu/Zn ratios should be given more caution. Meanwhile, the Cu/Zn ratio is a factor that can be adjusted by diet therapy. Further research is needed to establish the optimal value of the Cu/Zn ratio and to clarify the effectiveness of interventions aimed at lowering the Cu/Zn ratio, such as dietary Zn supplementation.

## References

[1] Chaudhuri A, Ghanim H, Arora P (2022). Improving the residual risk of renal and cardiovascular outcomes in diabetic kidney disease: a review of pathophysiology, mechanisms, and evidence from recent trials. Diabetes Obes Metab.

[2] Alicic RZ, Rooney MT, Tuttle KR (2017). Diabetic kidney disease: challenges, progress, and possibilities. Clin J Am Soc Nephrol.

[3] Alicic RZ, Johnson EJ, Tuttle KR (2018). Inflammatory mechanisms as new biomarkers and therapeutic targets for diabetic kidney disease. Adv Chronic Kidney Dis.

[4] Mora-Fernández C, Domínguez-Pimentel V, de Fuentes MM, Górriz JL, Martínez-Castelao A, Navarro-González JF (2014). Diabetic kidney disease: from physiology to therapeutics. J Physiol.

[5] González-Juanatey JR, Górriz JL, Ortiz A, Valle A, Soler MJ, Facila L (2023). Cardiorenal benefits of finerenone: protecting kidney and heart. Ann Med.

[6] Di Lullo L, Lavalle C, Scatena A, Mariani MV, Ronco C, Bellasi A (2023). Finerenone: questions and answers-the four fundamental arguments on the new-born promising non-steroidal mineralocorticoid receptor antagonist. J Clin Med.

[7] Chen W, Zheng L, Wang J, Lin Y, Zhou T (2023). Overview of the safety, efficiency, and potential mechanisms of finerenone for diabetic kidney diseases. Front Endocrinol (Lausanne).

[8] Olechnowicz J, Tinkov A, Skalny A, Suliburska J (2018). Zinc status is associated with inflammation, oxidative stress, lipid, and glucose metabolism. J Physiol Sci.

[9] Li B, Tan Y, Sun W, Fu Y, Miao L, Cai L (2013). The role of zinc in the prevention of diabetic cardiomyopathy and nephropathy. Toxicol Mech Methods.

[10] Hamasaki H, Kawashima Y, Yanai H (2016). Serum Zn/Cu ratio is associated with renal function, glycemic control, and metabolic parameters in Japanese patients with and without type 2 diabetes: a cross-sectional study. Front Endocrinol (Lausanne).

[11] Gembillo G, Labbozzetta V, Giuffrida AE (2022). Potential role of copper in diabetes and diabetic kidney disease. Metabolites.

[12] Ahmad S, Ärnlöv J, Larsson SC (2022). Genetically predicted circulating copper and risk of chronic kidney disease: a mendelian randomization study. Nutrients.

[13] Malavolta M, Piacenza F, Basso A, Giacconi R, Costarelli L, Mocchegiani E (2015). Serum copper to zinc ratio: relationship with aging and health status. Mech Ageing Dev.

[14] Takao T, Yanagisawa H, Suka M (2022). Synergistic association of the copper/zinc ratio under inflammatory conditions with diabetic kidney disease in patients with type 2 diabetes: the Asahi Diabetes Complications Study. J Diabetes Investig.

[15] Inoue S, Kondo Y, Yoshida H (2018). Evaluation of the reagent for measurement of Zinc ESPA・Zn Ⅱ. J Clin Lab Inst Reag.

[16] Abe A, Yamashita S, Noma A (1989). Sensitive, direct colorimetric assay for copper in serum. Clin Chem.

[17] Aderka D, Sorkine P, Abu-Abid S (1998). Shedding kinetics of soluble tumor necrosis factor (TNF) receptors after systemic TNF leaking during isolated limb perfusion. Relevance to the pathophysiology of septic shock. J Clin Invest.

[18] Biarnés J, Fernández-Real JM, Fernández-Castañer M, del Mar García M, Soler J, Ricart W (2005). Differential regulation of insulin action and tumor necrosis factor α system activity by metformin. Metabolism.

[19] Tanaka M, Matsuo K, Enomoto M, Mizuno K (2004). A sol particle homogeneous immunoassay for measuring serum cystatin C. Clin Biochem.

[20] Shlipak MG, Sarnak MJ, Katz R (2005). Cystatin C and the risk of death and cardiovascular events among elderly persons. N Engl J Med.

[21] Astor BC, Levey AS, Stevens LA, Van Lente F, Selvin E, Coresh J (2009). Method of glomerular filtration rate estimation affects prediction of mortality risk. J Am Soc Nephrol.

[22] Waheed S, Matsushita K, Sang Y, Hoogeveen R, Ballantyne C, Coresh J, Astor BC (2012). Combined association of albuminuria and cystatin C-based estimated GFR with mortality, coronary heart disease, and heart failure outcomes: the Atherosclerosis Risk in Communities (ARIC) Study. Am J Kidney Dis.

[23] Shlipak MG, Matsushita K, Ärnlöv J (2013). Cystatin C versus creatinine in determining risk based on kidney function. N Engl J Med.

[24] Horio M, Imai E, Yasuda Y, Watanabe T, Matsuo S (2013). GFR estimation using standardized serum cystatin C in Japan. Am J Kidney Dis.

[25] (2025). Health Japan 21 (the second term). https://www.nibiohn.go.jp/eiken/kenkounippon21/en/kenkounippon21/genjouchi.html#dai5_04.

[26] Yanagisawa H, Kawashima T, Miyazawa M, Ohshiro T (2016). Validity of the copper/zinc ratio as a diagnostic marker for taste disorders associated with zinc deficiency. J Trace Elem Med Biol.

[27] Kung WJ, Shih CT, Lee CH, Lin CC (2018). The divalent elements changes in early stages of chronic kidney disease. Biol Trace Elem Res.

[28] Mocchegiani E, Malavolta M, Lattanzio F (2012). Cu to Zn ratio, physical function, disability, and mortality risk in older elderly (ilSIRENTE study). Age (Dordr).

[29] Giacconi R, Costarelli L, Piacenza F (2017). Main biomarkers associated with age-related plasma zinc decrease and copper/zinc ratio in healthy elderly from ZincAge study. Eur J Nutr.

[30] Krolewski AS, Niewczas MA, Skupien J (2014). Early progressive renal decline precedes the onset of microalbuminuria and its progression to macroalbuminuria. Diabetes Care.

